# Comprehensive analysis of human monocyte subsets using full-spectrum flow cytometry and hierarchical marker clustering

**DOI:** 10.3389/fimmu.2024.1405249

**Published:** 2024-04-29

**Authors:** Chao Li, Maozhi Xiao, Suxia Geng, Yulian Wang, Lingji Zeng, Peilong Lai, Ying Gong, Xiaomei Chen

**Affiliations:** ^1^ Department of Hematology, Guangdong Provincial People’s Hospital (Guangdong Academy of Medical Sciences), Southern Medical University, Guangzhou, China; ^2^ Department of Laboratory Medicine, Guangdong Engineering and Technology Research Center for Rapid Diagnostic Biosensors, Nanfang Hospital, Southern Medical University, Guangzhou, China

**Keywords:** spectral flow cytometry, monocytes, tumor microenvironment (TME), immunophenotyping, immune checkpoints, t-distributed stochastic neighbor embedding (tSNE) analysis, mesenchymal stromal cells (MSCs)

## Abstract

**Introduction:**

Exploring monocytes’ roles within the tumor microenvironment is crucial for crafting targeted cancer treatments.

**Methods:**

This study unveils a novel methodology utilizing four 20-color flow cytometry panels for comprehensive peripheral immune system phenotyping, specifically targeting classical, intermediate, and non-classical monocyte subsets.

**Results:**

By applying advanced dimensionality reduction techniques like t-distributed stochastic neighbor embedding (tSNE) and FlowSom analysis, we performed an extensive profiling of monocytes, assessing 50 unique cell surface markers related to a wide range of immunological functions, including activation, differentiation, and immune checkpoint regulation.

**Discussion:**

This in-depth approach significantly refines the identification of monocyte subsets, directly supporting the development of personalized immunotherapies and enhancing diagnostic precision. Our pioneering panel for monocyte phenotyping marks a substantial leap in understanding monocyte biology, with profound implications for the accuracy of disease diagnostics and the success of checkpoint-inhibitor therapies. Key findings include revealing distinct marker expression patterns linked to tumor progression and providing new avenues for targeted therapeutic interventions.

## 1 Introduction

Monocytes represent a heterogeneous cell population of circulating leukocytes characterized by variations in phenotype and function (1). They play a pivotal role in coordinating both innate and adaptive immune responses to pathogens and endogenous stimuli (2, 3). Monocytes execute a spectrum of functions including phagocytosis, release of reactive oxygen species, secretion of cytokines and chemokines, recruitment of neutrophils, antigen presentation, and modulation of lymphocyte activity. Monocytes can be divided into three subsets based on the presence of the cell surface lipopolysaccharide (LPS) co-receptor CD14, and Fc-Gamma Receptor III (CD16). CD14^++^ CD16^−^ classical monocytes account for ~85% of circulating monocytes, intermediate cells (CD14^++^ CD16^+^) for ~5% and non-classical cells (CD14^-^ CD16^++^) for ~10% of circulating monocytes (4, 5). In the context of inflammation and tumor microenvironment (TME), there is a notable increase in monocyte populations (6). For instance, the ratio of classical monocytes is a sensitive and specific marker for Chronic Myelomonocytic Leukemia (CMML), differentiating it from Myelodysplastic Syndrome (MDS) (7), yet it does not distinguish CMML from atypical chronic myeloid leukemia (aCML) (8). This highlights the limitations of relying solely on CD14/CD16 and the necessity for exploring additional markers and reclassifying monocyte subgroups for more precise diagnostics

Based on their ability to regulate immune responses, Mesenchymal stromal cells (MSCs) are considered to be potential candidates for managing immune-mediated diseases in the context of immune therapy (9). Monocytes engage in extensive interactions with MSCs and other immune cells, facilitated by immune-related antigens, surface molecules, and regulatory factors (9, 10). These interactions are part of the complex communication network within the TME that influences immune responses and cancer progression and may offer potential targets for therapeutic interventions. These molecules, including costimulatory markers such as CD86, CD80, CD163, CD73, and HLA-DR (11–13), along with co-inhibitory receptors (e.g., PD-1, LAG-3), and cytokine/chemokine receptors (e.g., CX3CR1, CXCR5).

The costimulatory markers are integral to T cell biology, influencing the functional outcome of T cell receptor (TCR) signaling and dictating T cell function and fate (14). Investigating the co-stimulatory signals on monocytes enhances our understanding of their impact on the activation, proliferation, and function of immune cells, such as T cells and MSCs, mediated by monocytes. Delving into these signals involving monocytes uncovers the underlying mechanisms of immune responses, thus revealing potential mechanisms underlying disease progression and informing the development of effective strategies for disease prevention, diagnosis, and immunotherapy treatment. Moreover, costimulatory signals play a critical role in regulating the establishment and maintenance of immune tolerance (15, 16). Understanding their intricate involvement in immune tolerance mechanisms not only enhances our understanding of the mechanisms of a wide range of diseases, including autoimmune diseases, infectious diseases, tumors, and various other illnesses but also propels the development of innovative approaches to research on immune tolerance (14). The significance of thoroughly studied co-stimulatory signals and activation markers on monocytes, as essential components of antigen presentation cells, cannot be overstated.

In recent years, checkpoint inhibitors gained increasing recognition as promising therapeutic targets. Understanding checkpoints on monocytes can provide valuable insights into the mechanisms of interaction between monocytes and MSCs, and offer critical clues for the development of novel immunotherapy strategies. Only individual checkpoints on monocytes in tumors and diseases have been studied in previous studies, with a lack of comprehensive systematic investigation of checkpoints on monocytes reported (17–19). As the utilization of checkpoint inhibitor immunotherapies continues to expand within the realm of cancer research and clinical practice, there emerges a pressing requirement for assays capable of accurately characterizing the phenotypes of immune cells in patients (20). We offer a comprehensive panel to assess the expression of monocyte checkpoints, facilitating a thorough understanding of the immunomodulatory effects of monocytes in diseases and the identification of effective immunotherapy targets.

Cytokine/chemokine receptors, a class of membranous receptors, play pivotal roles in intercellular signaling, such as monocytes and MSCs, orchestrating fundamental biological processes containing immune response, inflammation, and cellular proliferation (21). A comprehensive understanding of monocyte characteristics requires an exploration of cytokine receptors. Insights gained from studying these receptors will aid researchers in understanding the intricate regulatory mechanisms of the immune system, offering valuable guidance for disease treatment and the development of immunotherapies.

Flow cytometry stands out as a highly powerful tool for unraveling the diversity and functional alterations of monocytes. This elucidation is crucial for comprehending their mechanisms and devising therapeutic strategies for diseases. Hence, we employ a multi-color spectral flow cytometry approach to investigate the panel of 50 markers in peripheral blood monocytes. By examining multiple costimulatory signals, activation markers, cytokine receptors, differentiation markers, and immune checkpoints, we aim to explore the surface marker expression profile of monocytes and dissect the differences among the three monocyte subgroups. This initiative facilitates the early detection of diseases, provides the target for immunotherapy, and enhances comprehension of the functions performed by monocytes.

## 2 Materials and methods

### 2.1 Blood donors and ethics statement

For screening experiments, peripheral venous blood samples (2 mL) were collected in BD K2-EDTA anticoagulant (BD Biosciences) by venipuncture from 20 healthy volunteers including 11 males (55%) and 9 females (45%). The average age for males is 48.7 (35-61) years old, and the females are 43.5 (16-63) years old (P=0.61).

This study received approval from the Ethics Committee of the Guangdong Provincial Hospital (Ethical Approval Number: KY-Z-2021-300-02). In strict accordance with the Declaration of Helsinki, we rigorously implemented informed consent procedures. Written consent, informed and comprehensive, was obtained from every participant enrolled in this study.

### 2.2 Flow cytometry sample preparation

Peripheral blood samples were collected with K2-EDTA as an anticoagulant, and mature red blood cells were lysed within 15 mins using 1 ml of 1x Lysing Buffer (BD Biosciences, San Jose, CA, USA) without any fixative. The resulting cell suspension was maintained in phosphate-buffered saline (PBS) containing 2% fetal bovine serum (GIBCO). The cell suspension was allocated into 4 test tubes, ensuring that the number of nucleated cells in each tube did not exceed 1×10^6^. Cells were stained with the following monoclonal antibodies (5 μL/sample) within 50μL BV buffer (BD), distributed evenly across the 4 tubes:

(1) BB515-conjugated CD45RA, PE-CY7-conjugated CD197(CCR7), PE-CF594-conjugated CD25, BV605-conjugated CD28, APC-R700-conjugated CD127 (IL-7Ra), APC-conjugated CD161, APC-R700-conjugated CD27, BV786-conjugated CD38; PE-conjugated CD57; BV421-conjugated CD69
(2) BC515-conjugated NKP44(CD336), PE-Dazzle594-conjugated NKG2A(CD159A), PE-Fire 810-conjugated CD86, BV605-conjugated CD80, R718-conjugated KIR3DL1(CD158E1), APC-conjugated CD163, APC-H7-conjugated HLA-DR, BV785-conjugated NKG2D(CD314); PE-conjugated KIR2DL1(CD158A), BV421-conjugated NKp46(CD335);
(3) BB515-conjugated CX3CR1, PE-Dazzle594-conjugated CD39, PE-Fire 810-conjugated CD194(CCR4), BV605-conjugated CD47, AF700-conjugated CD64, APC-conjugated CD34, APC-CY7-conjugated CD36, BV785-conjugated CD274(PD-L1); PE-conjugated CD73, BV421-conjugated CD123(IL-3R);
(4) BB515-conjugated CD278(ICOS), PE-Dazzle594-conjugated Tim-3(CD366), PE-Fire 810-conjugated TIGIT, BV605-conjugated CD152(CTLA-4), APC-R700-conjugated CD223(LAG-3), APC-conjugated CD272(BTLA), APC-CY7-conjugated CD117(c-kit), BV785-conjugated CXCR5(CD185); PE-conjugated CD294(CRTH2), BV421-conjugated CD279 (PD-1);

All cells were stained with the following shared monoclonal antibodies: PerCP-conjugated CD45; BV510-conjugated CD3; PerCP-eFluor 710-conjugated TCR-γδ; BV480-conjugated CD19; AF647-conjugated CD56; BV711-conjugated CD16; BV570-conjugated CD11b; efluor 450-conjugated CD11c; Spark Blue 550-conjugated CD14; FITC-conjugated CD15; Detailed antibody information can be found in **Table 1**. After staining, samples were incubated at room temperature for 30 minutes in the dark, then treated in 2 ml PBS containing 2% fetal bovine serum (GIBCO). After two consecutive washing steps, centrifugation was performed at 300 × g for 5 min before samples were collected on a Cytek Aurora/NL (Cytek Biosciences) using Cytek SpectroFlo software.

**Table 1:** Survey panel antibody reagents.

Target	Conjugate	PURPOSE	Clone	SOURCE
CD86	PE-Fire 810	Activation marker	IT2.2	Biolegend
HLA-DR	APC-H7	Activation marker	L243 (ASR)	BD
CD278(ICOS)	BB515	Activation marker	DX29 (RUO)	BD
CD38	BV786	Activation marker	HIT2 (RUO)	BD
CD11b	BV570	APCs marker	M1/70	BioLegend
CD19	BV480	B cell lineage	SJ25C1 (also known as SJ25-C1) (RUO)	BD
CD28	BV605	B cell lineage	CD28.2 (RUO)	BD
CD36	APC-CY7	B cell lineage	5-271	Biolegend
CD39	PE/Dazzle 594	B cell lineage	Duha59	Biolegend
CD123(IL-3R)	BV421	Basophils and pDCs cell lineage	6H6	Biolegend
NKG2D(CD314)	BV785	Co-stimulation molecule	1D11	Biolegend
CD80	BV605	Co-stimulation molecule	2D10.4 (also known as 2D10) (RUO)	BD
CD163	APC	Co-stimulation molecule	RM3/1	Biolegend
CD73	PE	Co-stimulation molecule	AD2 (RUO)	BD
CD11c	efluor 450	DCs marker	3.9	eBioscience
CD27	APC-H7	Differentiation marker	M-T271 (RUO)	BD
CD57	PE	Differentiation marker	NK-1 (RUO)	BD
CD279 (PD-1)	BV421	Exhaustion marker	EH12.1 (also known as EH12) (RUO)	BD
CD274(PD-L1)	BV785	Exhaustion marker	29E.2A3	Biolegend
TIGIT	PE-Fire 810	Exhaustion marker	A15153G	Biolegend
Tim-3(CD366)	PE-Dazzle 594	Exhaustion marker	F38-2E2	Biolegend
CD272(BTLA)	APC	Exhaustion marker	J168-540 (RUO)	BD
CD152(CTLA-4)	BV605	Exhaustion marker	BNI3	Biolegend
CD223(LAG-3)	APC-R700	Exhaustion marker	T47-530 (RUO)	BD
CD34	APC	Hpc marker	8G12 (also known as HPCA2) (ASR)	BD
CD117(c-kit)	APC-CY7	ILC subsets marker	104D2	Biolegend
CD47	BV605	Macrophagocyte marker	G18-145 (RUO)	BD
CD161	APC	MAIT cell marker	DX12 (RUO)	BD
CD45RA	BB515	Memory cell marker	HI100 (RUO)	BD
CX3CR1	BB515	Migration marker	2A9-1 (RUO)	BD
CD194(CCR4)	PE-Fire 810	Migration marker	L291H4	Biolegend
CD294(CRTH2)	PE	Migration marker	BM16 (RUO)	BD
CD14	Spark Blue 550	Monocytes cell marker	63D3	BioLegend
CD15	FITC	Myeloid cell lineage	MMA (ASR)	BD
CD197(CCR7)	PE-CY7	Naïve cell marker	3D12 (RUO)	BD
CD64	AF700	Neutrophile marker	10.1 (RUO)	BD
NKG2A(CD159A)	PE-Dazzle594	NK cell lineage	S19004C	Biolegend
KIR2DL1(CD158A)	PE	NK cell lineage	HP-MA4 (RUO)	BD
KIR3DL1(CD158E1)	R718	NK cell lineage	DX9 (RUO)	BD
NKP44(CD336)	BB515	NK cell lineage	p44-8 (RUO)	BD
NKp46(CD335)	BV421	NK cell lineage	9E2/NKp46 (also known as 9-E2) (RUO)	BD
CD56	AF647	NK cell lineage/NKT-like cell	B159 (RUO)	BD
CD16	BV711	NK cell lineage/NKT-like cell	3G8 (RUO)	BD
CD25	PE-CF594	Regulatory T cell lineage	M-A251 (RUO)	BD
CD127 (IL-7Ra)	APC-R700	Regulatory T cell lineage	HIL-7R-M21 (RUO)	BD
CD3	BV510	T cell lineage	UCHT1 (also known as UCHT-1; UCHT 1) (RUO)	BD
TCR-γδ	PerCP-eFluor 710	T cell lineage	B1.1	eBioscience
CXCR5(CD185)	BV785	Tfh marker	J252D4	Biolegend
CD69	BV421	Tissue residency marker	FN50 (also known as FN 50) (RUO)	BD
CD45	PerCP	White blood cell lineage	HI30	BioLegend

### 2.3 Flow cytometry data analysis

Flow cytometry data was analyzed using Flowjo (BD Biosciences, San Jose, CA, USA) (Version 10.6.2, https://www.flowjo.com/). The expression pattern of each marker of 4 panels was assessed in the monocytes. FlowSOM, utilizing Self-Organizing Maps (SOMs), based on marker expression phenotype, was employed to assign all individual cells into clusters and metaclusters (group of clusters) (22), FlowSOM was used with default settings unless otherwise noted.

## 3 Results

### 3.1 Full spectrum flow cytometry measures the subsets of human peripheral blood cells

**Figure 1:**
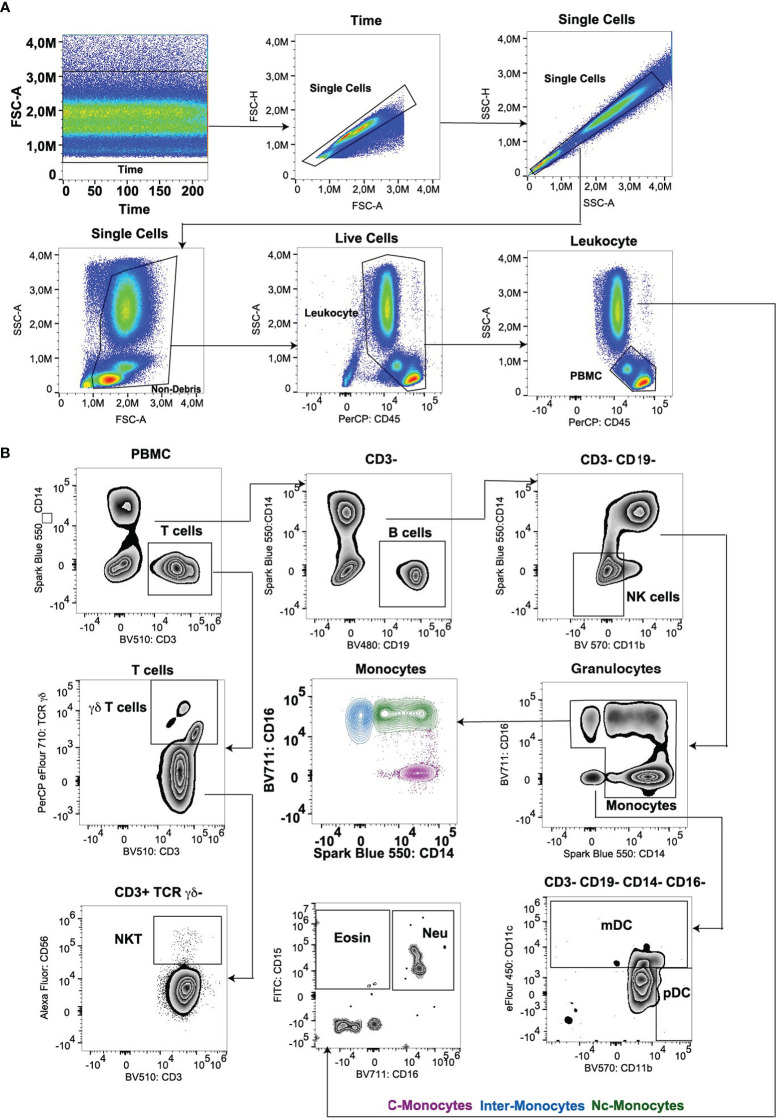
**(A)** Gating strategy for lineage panel of peripheral blood leukocyte subsets. **(B)** Gating main PBMC cell subsets (T, B, NK, monocytes, and DCs) and Myeloids (eosinophil and neutrophil). C-Mono, classical monocytes; Inter-Mono, intermediate monocytes; Nc-Mono, non-classical monocytes; Eosin, Eosinophil; Neu, neutrophil.

Utilizing full spectrum flow cytometry, we selected the major immune cell lineage markers listed in **Table 1** to establish a multicolor panel for the comprehensive immuno-phenotyping of human peripheral blood samples. Duplexes were excluded from the total cell population using FSC-H versus FSC-A plots. Mononuclear cells were gated using side scatter (SSC) versus CD45 dot plots after the exclusion of debris. Initial gating procedures involved: (i) identifying pan-leukocytes through CD45 expression, (ii) excluding deceased cells and debris based on FSC/SSC attributes, (iii) eliminating doublets and aggregates using distinct FSC signals, and, if necessary, (iv) excluding counting bead contamination. Subsequently, PBMCs were isolated from granulocytes utilizing position-based gating strategies, which allowed for the precise separation of these distinct cell populations (**Figure 1A**). By strategically positioning gates, PBMCs were effectively distinguished, facilitating subsequent analyses focused on this particular cell subset by not gate strategies. T cells were isolated from granulocytes utilizing a bivariate CD3/CD14 plot. CD3^+^ T cells are subsequently divided into αβ/γδ T cells by CD3/TCR γδ plot. The NKT cells were divided from the αβ T cells by CD56/CD3 plot. Within the CD3^+^ T cell-excluded PBMC compartment, CD19^+^ B cells are defined. The bulk of CD3/CD19 double-negative cells contain CD11b^-^/CD14^-^ natural killer (NK) cells expressing CD56 (and for the most part CD16) as well as populations of CD11b/CD14 single- or double-positive cells. This latter fraction comprises monocyte populations that express CD14 and/or CD16 as well as a population of CD14/CD16 double-negative cells. The CD14^-^/CD16^-^ fraction contains both CD11c^+^ myeloid dendritic cells (mDCs) and CD11c^-^/CD11b^+^ plasmacytoid dendritic cells (pDCs). CD15+ granulocytes are subsequently divided into CD16^+^ neutrophils and CD16^-^ eosinophils (**Figure 1B**).

### 3.2 Comprehensive analysis of multiple myeloid markers in human monocyte subsets

**Figure 2:**
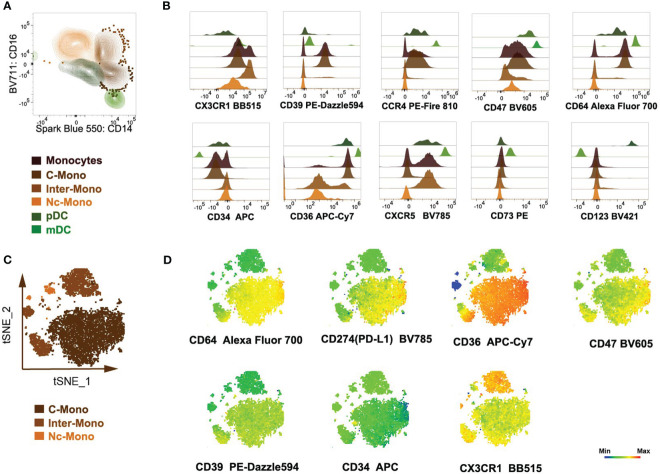
Analysis of peripheral blood monocytes cells of myeloid markers and chemokine receptors. **(A)** Monocytes and DCs were isolated utilizing a bivariate CD14/CD16 plot. **(B)** Histogram overlays depicted the expression of myeloid markers and chemokine receptor markers within monocytes and DCs population. **(C, D)** Semi-automated analysis of flow cytometry data by tSNE. Scale bars of the tSNE plot represented color-coded scaled fluorescence intensity or cell count levels.

In our investigation of monocyte subsets utilizing an extensive panel of surface markers, including CD14, CD16, CD64, CD123, CD34, CD36, CD123, CD47, CX3CR1, CCR4, CXCXR5, CD39, and CD73, we observed a diversity of phenotypic profiles within the monocyte population. While the classical (CD14^++^CD16^-^), intermediate (CD14^++^CD16^+^), and non-classical (CD14^-^CD16^++^) subsets were readily discernible, our multi-marker approach unveiled transitional populations exhibiting intermediate phenotypes (**Figure 2C**).

Simultaneously, a comparison was made on DCs (**Figure 2A**). Expression levels of CD64, CD123, and CD34 significantly contributed to delineating monocyte subsets and their functional attributes. Elevated expression of CD64 was associated with classical monocytes and pDCs, and correlated with enhanced phagocytic activity and pro-inflammatory cytokine secretion (**Figures 2B, D**). Conversely, higher levels of CD34 expression were observed in intermediate and non-classical monocytes, indicative of anti-inflammatory and tissue-resident properties (**Figures 2B, D**). Elevated CD123 expression on monocytes has been linked to certain pathological conditions, such as hematological malignancies (23) and autoimmune disorders (24), highlighting its potential as a biomarker for disease monitoring and therapeutic targeting, while normal monocytes exhibited a lack of CD123 expression (**Figures 2B, D**). The expression of CD36, CD47, and CX3CR1 delineated distinct functional states within monocyte subsets. CD36 expression is associated with enhancing lipid uptake and metabolic functions (25) predominantly expressed in classical monocytes. CD36^hi^ monocytes may contribute to the peripheral development of Foxp3^+^ T-bet^+^ T cells with regulatory functions (26). While CD47 was expressed in normal DCs (mDCs and pDCs subsets) associated with immune evasion and anti-phagocytic properties (**Figures 2B, D**), in autoimmune diseases and inflammatory-related diseases, CD47 is highly expressed in monocytes (27, 28). CX3CR1 expression correlated with tissue homing and surveillance functions, suggesting a role in intermediate monocyte subset trafficking and localization within peripheral tissues (**Figures 2B, D**).

Analysis of chemokine receptor expression revealed differential patterns among monocyte subsets. While CCR4 expression was enriched in classical monocytes, CXCR5 expression was predominantly observed in intermediate monocytes (**Figures 2B, D**), implicating differential chemotactic responses and migratory behaviors within the monocyte pool. CD73 is expressed on approximately 50-70% of lymphoid cells, whereas it is not expressed on normal monocytes. CD39 is expressed on >90% of monocytes (13), mostly on classical monocytes (**Figure 2D**).

### 3.3 Co-stimulatory and activation markers are differently expressed in monocyte subpopulations

**Figure 3:**
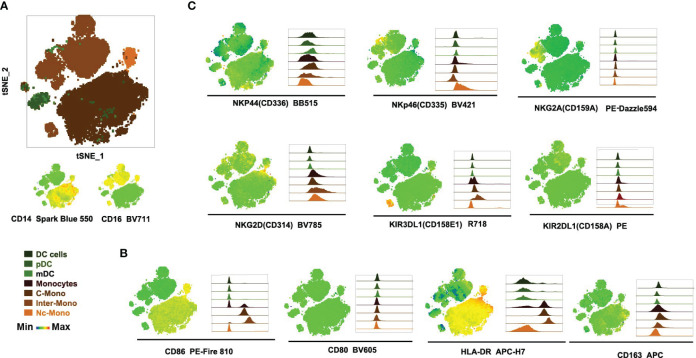
Expression of activating receptors and co-stimulation markers by monocyte subsets. **(A)** Three monocyte subsets clustered by tSNE. **(B, C)** Visualization of co-stimulation and activation markers in monocyte subsets, with the left side showing tSNE plots and the right side showing histogram plots.

Through the utilization of t-distributed stochastic neighbor embedding (tSNE) analysis, distinct subsets including classical monocytes (C-Mono), intermediate monocytes (Inter-Mono), non-classical monocytes (Nc-Mono), pDCs, and mDCs were delineated (**Figure 3A**). We compared the co-stimulatory and activation markers of different monocyte subpopulations. HLA-DR, CD163, CD80, and CD86 revealed intricate patterns of expression indicative of functional diversity within the monocyte subpopulations (**Figure 3B**). Monocytes exhibited heterogeneous expression patterns of the co-stimulatory molecules CD86, critical for antigen presentation and T cell activation. Classical and intermediate monocytes displayed high expression levels of CD86, suggesting an activated phenotype involved in antigen presentation, with the up-regulated expression on intermediate monocytes correlating with disease severity and impacting immunomodulatory processes (11, 29). CD80 expression did not exhibit subset-specific variations suggesting that its expression may be regulated by common mechanisms across all monocyte subsets (**Figure 3B**). Additionally, the expression of HLA-DR varied among monocyte subsets, with classical monocytes showing high levels of expression, indicative of their involvement in antigen presentation and immune surveillance (**Figure 3B**). The presence of CD163 on classical monocytes suggests their anti-inflammatory properties and potential involvement in phagocytic clearance and inflammation resolution (12) (**Figure 3B**). Furthermore, to assess the specificity of our monocyte sorting process, we utilized negative controls targeting markers specifically associated with NK cells (NKp44, NKp46, NKG2A, KIR2DL1, NKG2D, and KIR3DL1) (**Figure 3C**). These negative controls confirmed the absence of NK cell contamination in the sorted cell population, ensuring the purity and specificity of the isolated monocyte subset.

### 3.4 Checkpoint markers expression pattern analysis between monocyte subsets

**Figure 4:**
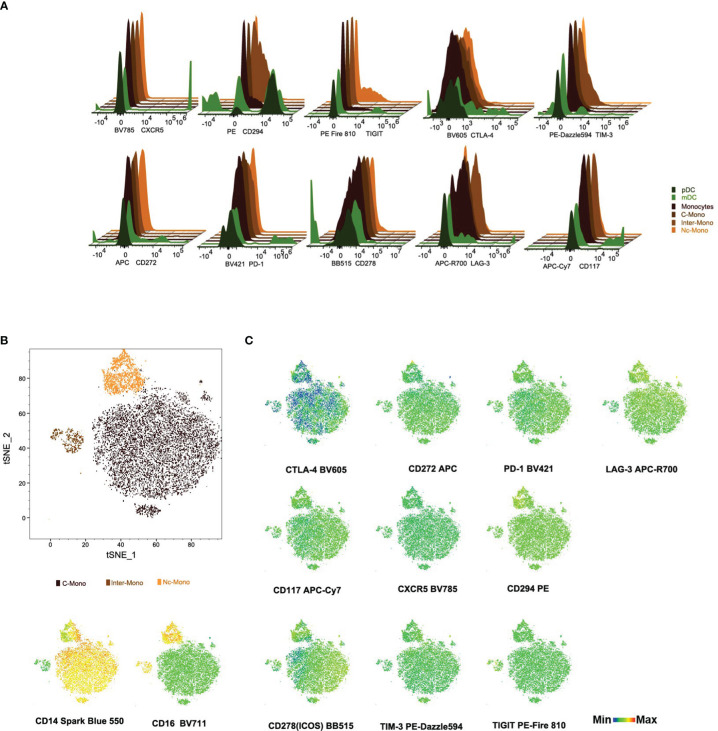
Immune checkpoint molecule profiles on subsets of monocytes and dendritic Cells **(A)** Histogram plots illustrate checkpoint markers distribution among monocytes and DCs subsets. **(B, C)** tSNE visualization of checkpoint molecule expression and distribution in monocyte subsets from PBMCs. This figure maps 12 checkpoint molecules and cell counts in PBMC-derived monocytes, with scale bars indicating fluorescence and distribution metrics.

Next, the expression profiles of immune checkpoint molecules and chemokine receptors on monocytes and DCs were investigated, including CTLA4, CD272, CRTH2/CD294, LAG-3, ICOS/CD278, TIM-3, TIGIT, PD-1, CD117, CXCR5, elucidated the nuanced heterogeneity and regulatory potential within the monocyte and DCs population. CD294 exhibits a subdued expression within the Inter-mono subset while manifesting discernible expression levels within mDCs and pDCs subsets. CD117, CXCR5, and PD-1 are mildly expressed within the mDCs subset but remain absent in the pDCs subset as well as across all monocyte subsets (**Figure 4A**), with PD-1 highly expressed in monocytes in patients with hepatocellular carcinoma, presenting a preference toward M2 polarization and had a deficiency in supporting CD8 T cells (18). CTLA^-^4 demonstrates ubiquitous expression across all DC subsets, contrasting with its absence within all monocyte subsets (**Figure 4A**). Regarding CD278/ICOS, there is a distinctive distribution observed within the C-Mono subset, where a minority of cells exhibit diminished levels of CD278/ICOS expression (**Figure 4C**). TIGIT displays a scarce expression within a limited population of Nc-Mono cells (**Figure 4A**), while decreased expression of TIGIT on CD14 ^+^ monocytes correlates with clinical features and laboratory parameters of patients with primary Sjögren’s syndrome (19). Furthermore, we observed that co-inhibitory molecules and exhaustion (CD272, LAG-3/CD223, and TIM-3) were not differently expressed on three monocyte subsets under normal physiological conditions (**Figures 4A, C**), although increased Tim-3^+^ monocytes/macrophages are associated with disease severity in patients with IgA nephropathy (30). Activated platelets promote increased monocyte expression of CXCR5 through prostaglandin E2-related mechanisms and enhance the anti-inflammatory effects of CXCL13 (21), whereas CXCR5 is upregulated in Ly6C^low^ inflammatory monocyte cells from all mice, presenting anti-inflammatory/atherogenic features (31). Although the expression of CD294 was low, it was specifically expressed by the non-classical monocyte subset at the RNA level (32). CD117, a cytokine receptor expressed on the surface of hematopoietic stem cells with a likely role in cell survival, proliferation, and differentiation (33), was lacking in three monocyte subsets. These findings underscore the diversity and regulatory complexity of monocytes in immune homeostasis and pathology, emphasizing the need for further investigation into the functional implications of immune checkpoint expression in monocyte-mediated immune responses.

### 3.5 Hierarchical clustering reveals hierarchy of markers discriminating monocyte subsets

**Figure 5:**
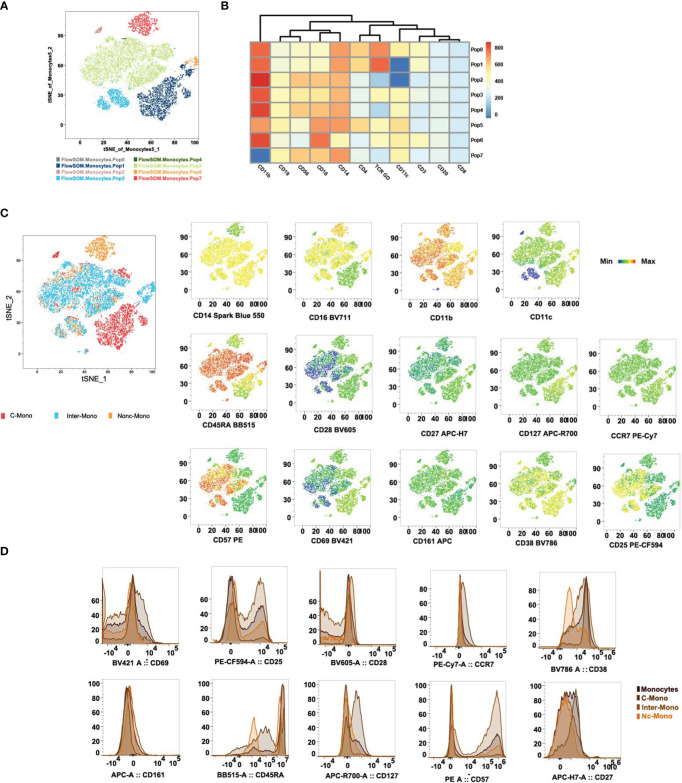
Differential expression of co-stimulatory, activation, and terminal differentiation/exhaustion markers cross monocyte subpopulations. **(A)** Visualization by tSNE reveals distinct monocyte populations, with the PhenoGraph algorithm identifying 8 unique cell clusters. Each cluster is visualized in a different color. The ClusterExplorer algorithm quantifies cell counts within these PhenoGraph clusters and facilitates the generation of a heatmap to examine protein expression patterns across each identified cluster[36]. **(B)** tSNE plots delineate the distribution of phenotypic markers among C-Mono, Inter-Mono, and Nc-Mono. **(C)** Histograms detail the distribution of phenotypic markers within monocyte subsets, highlighting differential expression profiles that distinguish monocyte subsets, shown are monocyte, C-Mono, Inter-Mono, and Nc-Mono. C-Mono, classical monocytes; Inter-Mono, intermediate monocytes; Nc-Mono.

tSNE and FlowSOM algorithms were applied to clustering to categorize monocytes based on 11 lineage markers, resulting in 7 clusters (**Figures 5A, B**). Subsequently, using CD14, CD16, CD11c, and CD11b, we delineated three monocyte subsets (**Figure 5C**). Our analysis unveiled distinct expression patterns of investigated markers (CD28, CD27, CD45RA, CD127, CD57, CCR7, CD69, CD161, CD38, and CD25) across classical, non-classical, and intermediate monocyte subsets (**Figures 5C, D**). Classical monocytes showed high CD28 and CD27 expression, suggesting antigen presentation and T-cell co-stimulation. Non-classical monocytes displayed elevated CD45RA expression and reduced CD127 expression, indicative of a mature and tissue-resident phenotype. CD57 expression was predominantly in intermediate monocytes, suggesting a terminal differentiation. CCR7 expression was weak in classical monocytes, implying similar migratory ability across subpopulations (**Figures 5C, D**). CD69 expression was moderate in classical and non-classical monocytes but low in intermediates, potentially impacting T-cell differentiation. The absence of CD161 was observed across all monocyte subsets. CD38 was expressed on classical and intermediate monocytes mostly in healthy volunteers, while CD25 was expressed on intermediate monocytes. These findings shed light on monocyte subset diversity and activation in various physiological and pathological contexts.

## 4 Discussion

The critical role of monocytes within the TME presents a nuanced landscape for understanding cancer progression and the efficacy of immunotherapies. Our study’s exploration of monocyte subsets through spectral flow cytometry has revealed significant insights into the complex dynamics at play within the TME. The differential expression profiles of classical and non-classical monocytes, particularly in the context of CMML (8) and MDS (34), underscore the potential and limitation of these cells as both diagnostic biomarkers and therapeutic targets.

In this study, we utilized advanced dimensionality reduction techniques, including FlowSom and t-SNE, to quantify forty key immunological cell surface markers in parallel, enabling precise categorization and role delineation of monocyte subsets across different pathological contexts. The identification of specific surface markers related to co-stimulation, differentiation, and immune checkpoint modulation in monocytes enriches our understanding of their immunoregulatory functions. Notably, the engagement of monocytes in antigen presentation and the modulation of T cell activity highlight their pivotal role in orchestrating anti-tumor immunity. The variation in the expression of markers such as CD25, CD28, and CD127, which indicate potential interactions between monocytes and T cells, could significantly influence T cell activation and lineage specification, ultimately affecting the immune response to tumors (35–38). Recent evidence suggests that reverse signaling involving ICOSL is also important in directing the differentiation of monocyte-derived cells (39). Furthermore, the expression of HLA-DR, CD80, and CD86 underscores monocytes’ capability in antigen processing and presentation, crucial for the potentiation of adaptive immunity (11, 40–42). HLA-DR serves as an important immunological marker in both diagnostic and prognostic settings. For instance, decreased HLA-DR expression levels on monocytes are associated with immunosuppression and can be observed in conditions such as sepsis or after hematopoietic stem cell transplantation (43). Additionally, CD38 and CD161 presence suggests roles in modulating inflammatory responses and NK cell functionalities, respectively (44, 45). The detection of CD127 (IL-7Ra) further points to monocytes’ sensitivity to IL-7 signals, essential for their survival and operational integrity (46). Moreover, CX3CR1 and CXCR5 expressions imply their engagement in site-specific migration and homing, critical for targeted recruitment to inflamed and lymphoid tissues (47, 48). Expression patterns of CD39 and CD73 indicate monocytes’ involvement in adenosine-dependent immune regulation, influencing inflammation resolution and tissue repair mechanisms (13). Moreover, the presence of immune checkpoints on monocytes within the TME, such as PD-1 and LAG-3, points to a complex mechanism of immune evasion employed by tumors. The expression of these checkpoints suggests that monocytes could be manipulated by tumor cells to create an immunosuppressive environment conducive to cancer progression (20). This insight opens new avenues for targeting the monocyte-mediated immunosuppression in cancer therapy, potentially through the use of checkpoint inhibitors designed to reactivate the anti-tumor immune response (17). Additionally, our findings emphasize the importance of the TME in shaping the phenotypic and functional properties of monocytes. The interaction between monocytes and other cells within the TME, including MSCs (9, 10) and tumor cells, further complicates the immune landscape. This interaction, mediated through a network of cytokines, chemokines, and cell surface molecules, could provide critical clues for developing strategies to manipulate the TME in favor of more effective cancer therapies.

Recent studies, including those by Thomas et al. (5) and Hoffmann et al. (49), have advanced our understanding of monocyte heterogeneity by delineating subsets based on surface marker expression. Classical monocytes are distinguished by high expression of CD14, CD36, and CCR2, while intermediate monocytes show elevated HLA-DR along with CD14, CD16, CD11c, and CD36. Non-classical monocytes are primarily identified by CD16 and CD11c, featuring lower HLA-DR levels. Novel markers such as BLTR1, CD35, and CD38 for classical; CD39, CD275, and CD305 for intermediate; and CD29 and CD132 for non-classical monocytes further refine these categorizations. Another study used multiparameter flow cytometry in the evaluation of myelodysplasia, SSC^int^/CD45^hi^CD33^hi,^ and/or CD14^hi^ used to gate the monocytes (50).

Our findings corroborate and expand upon this framework. Specifically, classical monocytes exhibit a broad marker profile including CD14, CD64, and notably CD38, which, alongside CD86, is significantly elevated compared to intermediate and non-classical monocytes. This differential expression diminishes as monocytes mature into the non-classical subset, marked by a distinctive downregulation of CD38 and CD86, highlighting their evolving functional capacities. CD69 emerges as a pivotal marker, with moderate expression in classical and non-classical subsets contrasting its notable absence in intermediate monocytes. This variation underscores CD69’s role in immune modulation, from influencing regulatory T cell differentiation via the *Jak3/Stat5* pathway (51) to its association with Alzheimer’s disease pathology (52). Additionally, the absence of CCR7 in monocytes disrupts migration and facilitates immunosuppression, leading to conditions such as chronic cutaneous leishmaniasis (53). Further, our analysis reveals a nuanced view of CD161 and CD25 (IL-2R α subunit) expressions. While CD161 is generally absent in healthy monocytes, its presence increases upon immune stimulation, indicating a responsive adaptation to immune challenges (54). CD25, indicative of T cell activation marker (55), shows elevated expressions in intermediate monocytes under health and disease states, linking it to thrombosis and *JAK2V617F* mutation in myeloproliferative neoplasms (35). The monocyte can be considered a maestro within the TME in both solid tumors and hematologic malignancies, moreover, the applications of checkpoint inhibitors, such as PD-1, CTLA-4, and BTLA, have emerged as a new upsurge of tumor immunotherapy. The new findings can not only detect the evolution of monocytes but also monitor changes in the expression of checkpoints (56–58). Together, these findings illustrate the complex expression patterns of co-stimulatory, activation, and exhaustion markers. Our study highlights how the TME intricately influences monocyte functions, emphasizing the complex interplay between monocytes, MSCs, and tumor cells. Through detailed analysis of co-stimulatory signals and activation markers, we underline monocytes’ crucial roles in antigen presentation and their potential in shaping therapeutic strategies for cancer, autoimmune, and inflammatory diseases. This research enriches our understanding of monocyte biology, paving the way for novel immunotherapeutic interventions.

The variance observed between our findings and previous research stems from the utilization of freshly collected peripheral blood samples, contrasting with the inherent limitations associated with frozen peripheral blood mononuclear cell preparations (5). Our study benefits from the advantage of utilizing fresh, non-cryopreserved samples, allowing for a more accurate representation of cellular phenotypes. By exploring certain surface markers not previously investigated, we offer novel insights into immune function and cellular interactions, enriching our understanding of monocyte biology. Moreover, this study may represent one of the first comprehensive and systematic assessments of immune checkpoint expression across monocyte subsets. Despite minimal differences observed among the three monocyte subsets, our findings enhance our comprehension of monocyte biology and present potential avenues for therapeutic intervention across various disease states, including autoimmune disorders, infectious diseases, and cancer.

However, this research acknowledges several limitations. While we explored diverse immune-relevant cell surface markers on monocytes using spectral flow cytometry, our focus remained primarily on expression patterns and their associations with immune function, without delving deeply into functional implications or disease-specific roles. Therefore, further investigations are warranted to fully characterize monocyte subsets and their interactions in immune regulation and disease pathology. Additionally, comprehensive assessments of immune checkpoint molecules across monocyte subsets in various diseases are imperative to understand their functional implications fully. As the field of monocyte biology continues to evolve rapidly, there is potential for the discovery of additional markers and mechanisms underlying immune regulation, which could further enhance our understanding of immune responses and disease processes.

## Data availability statement

The original contributions presented in the study are included in the article/supplementary material. Further inquiries can be directed to the corresponding authors.

## Ethics statement

The studies involving humans were approved by the Ethics Committee of the Guangdong Provincial Hospital (Ethical Approval Number: KY-Z-2021-300-02). The studies were conducted in accordance with the local legislation and institutional requirements. The participants provided their written informed consent to participate in this study.

## Author contributions

CL: Formal analysis, Visualization, Writing – original draft, Writing – review & editing. MX: Formal analysis, Investigation, Writing – original draft. SG: Data curation, Writing – original draft. YW: Software, Writing – original draft. LZ: Investigation, Writing – original draft. PL: Supervision, Writing – original draft. YG: Conceptualization, Methodology, Writing – review & editing, Writing – original draft. XC: Conceptualization, Project administration, Resources, Supervision, Writing – review & editing, Writing – original draft.
